# Targeting host-virus interactions: in silico analysis of the binding of human milk oligosaccharides to viral proteins involved in respiratory infections

**DOI:** 10.1038/s41598-024-54624-6

**Published:** 2024-02-19

**Authors:** Anees Ahmed Mahaboob Ali, Adarsh Vishal, Everette Jacob Remington Nelson

**Affiliations:** Gene Therapy Laboratory, Department of Integrative Biology, School of Bio Sciences and Technology, Vellore Institute of Technology, Vellore, 632 014 India

**Keywords:** Human milk oligosaccharides, Respiratory viral infections, Host-virus interactions, Molecular docking, MD simulations, Antiviral therapy, Computational biology and bioinformatics, Drug discovery, Microbiology

## Abstract

Respiratory viral infections, a major public health concern, necessitate continuous development of novel antiviral strategies, particularly in the face of emerging and re-emerging pathogens. In this study, we explored the potential of human milk oligosaccharides (HMOs) as broad-spectrum antiviral agents against key respiratory viruses. By examining the structural mimicry of host cell receptors and their known biological functions, including antiviral activities, we assessed the ability of HMOs to bind and potentially inhibit viral proteins crucial for host cell entry. Our in silico analysis focused on viral proteins integral to host-virus interactions, namely the hemagglutinin protein of influenza, fusion proteins of respiratory syncytial and human metapneumovirus, and the spike protein of SARS-CoV-2. Using molecular docking and simulation studies, we demonstrated that HMOs exhibit varying binding affinities to these viral proteins, suggesting their potential as viral entry inhibitors. This study identified several HMOs with promising binding profiles, highlighting their potential in antiviral drug development. This research provides a foundation for utilizing HMOs as a natural source for designing new therapeutics, offering a novel approach in the fight against respiratory viral infections.

## Introduction

**Figure 1:**
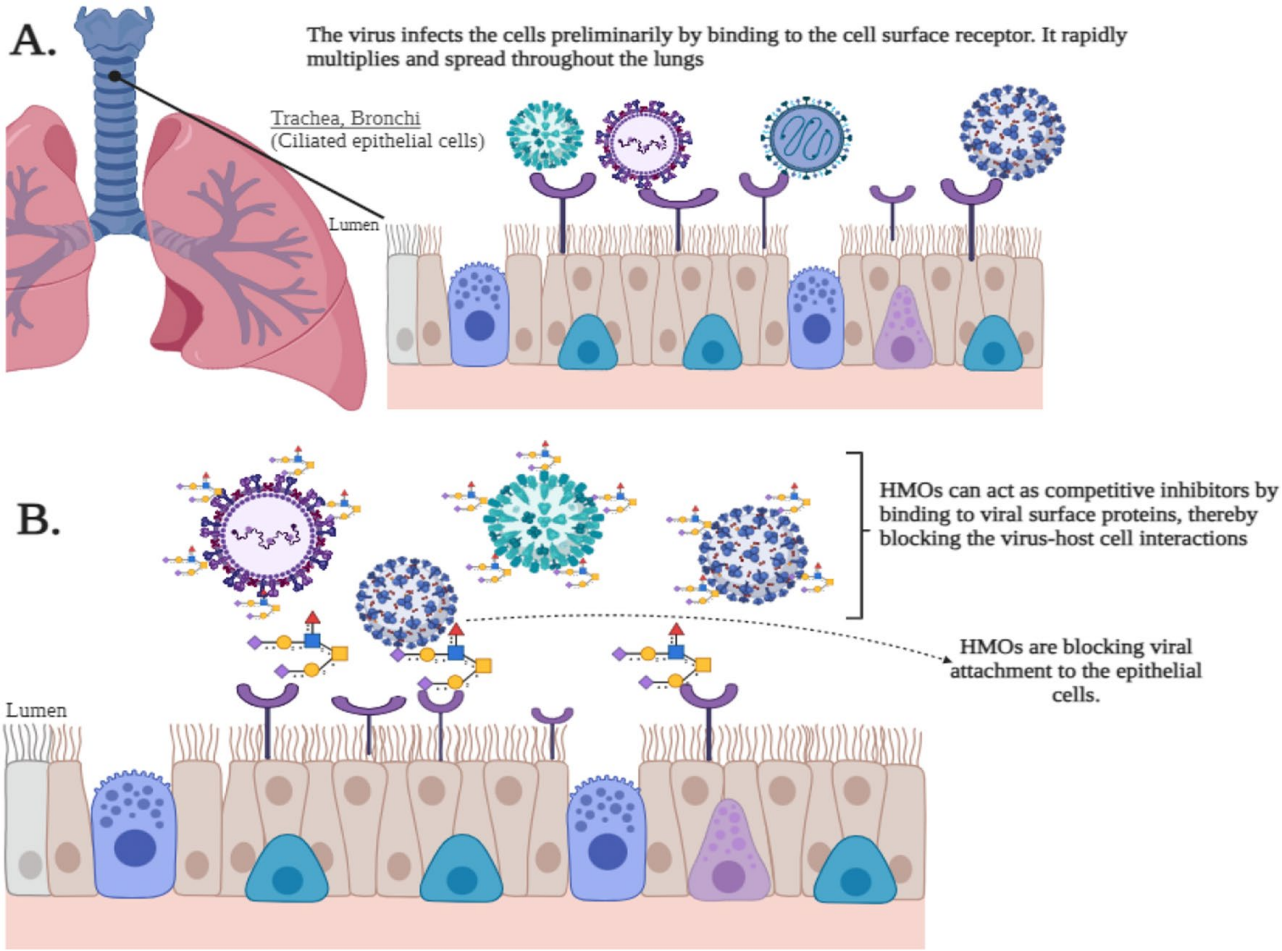
Mechanisms of viral infection and inhibition in the respiratory tract. (**A**) Viral entry and infection in the respiratory epithelium. This illustrates the process of viral infection in the respiratory tract. The virus initially binds to cell surface receptors on the ciliated epithelial cells lining the trachea and bronchi. Post-attachment, the virus enters the cells, replicates, and subsequently releases new viral particles, which further infect adjacent cells leading to the infection spreading throughout the lungs. (**B**) Inhibition of viral attachment by human milk oligosaccharides (HMOs). The mechanism by which HMOs impede viral infection is depicted. HMOs function as competitive inhibitors by attaching to viral surface proteins, thereby preventing the virus from interacting with host cell receptors. This blockage stops the virus from attaching to the epithelial cells which inhibits subsequent infection and spread within the respiratory tract. (Created with BioRender.com).

Respiratory viral infections pose a significant threat to public health worldwide. The impact of these viruses is accentuated by the continuous emergence of novel pathogens and the resurgence of existing pathogens, thereby making the development of effective antiviral therapies a crucial scientific and medical endeavor^1,2^. In this context, this study was focused on exploring human milk oligosaccharides (HMOs) as potential broad-spectrum antiviral agents for respiratory viruses. HMOs are complex carbohydrates uniquely found in human milk that are characterized by their remarkable structural diversity^3,4^, mirroring the complexity of glycan structures present on human epithelial cells. This positions HMOs as potential competitive inhibitors capable of binding to viral surface proteins, thus impeding virus-host cell interactions critical for their entry^5–8^, paving the way for investigating their role in antiviral strategies. Recent advances in synthesizing HMOs have enabled their production at a scale suitable for research and potential therapeutic use. Synthetic biology and enzymatic synthesis methods have been developed allowing the production of specific HMOs in quantities sufficient for detailed scientific investigations^9–11^. This development is significant as the natural extraction of these oligosaccharides from human milk is limited and not feasible for large-scale applications. The availability of synthesized HMOs has also led to their incorporation into infant formulas and nutritional supplements, reflecting their growing presence on the market in recognition of their health benefits^12^. This study relies on the hypothesis that HMOs can act as competitive inhibitors by binding to viral surface proteins thereby blocking the virus‒host cell interactions essential for viral entry and propagation (Fig. 1).

To evaluate this hypothesis, we selected key viral proteins that play pivotal roles in the pathogenic mechanisms of various respiratory infections, which include the hemagglutinin protein of influenza A virus, the fusion proteins of respiratory syncytial virus (RSV) and human metapneumovirus (HMPV), and the spike protein of SARS-CoV-2. These proteins are critical for the viruses’ ability to attach to and fuse with host cells, making them prime targets for potential inhibition by HMOs^13–16^. These viruses were selected based on their significant impact on public health, diversity in their mechanisms of infection, representation of different virus families, and relevance to current research and clinical context. This approach comprehensively evaluates the potential efficacy of HMOs in the treatment of various respiratory illnesses ranging from common infections to life-threatening pandemics. The chosen HMOs, such as 2′-fucosyllactose (2′-FL), lacto-N-neotetraose (LNnT), 3′-sialyllactose (3′-SL), lacto-N-hexaose (LNH), and lacto-N-fucopentaose III (LNFP3), represent a range of structurally variant oligosaccharides in human milk. This diversity is crucial because different structures may interact differently with viral proteins, offering a broader understanding of potential antiviral mechanisms. Additionally, their chemical properties, such as stability and solubility, make them feasible candidates for further pharmaceutical exploration. We employed an in silico approach utilizing molecular docking and simulation studies to investigate the interactions between selected HMOs and viral proteins. Molecular docking allows us to predict the binding affinities of HMOs for viral proteins providing insights into their potential efficacy as inhibitors. Subsequent molecular dynamics (MD) simulations offer a deeper understanding of the stability and behavior of these HMO-protein complexes, which is critical for evaluating their therapeutic potential. Molecular docking and MD simulations serve as foundational in silico tools for the discovery of molecules that may interfere with host-virus interactions. Our study employs these methods to identify HMOs with the potential to bind and stabilize key viral proteins, which are crucial for viral attachment and entry into host cells. While our findings are promising, we acknowledge that these computational predictions require validation through experimental approaches to confirm the biological efficacy of these interactions. Therefore, we consider these in silico techniques as part of an integrated approach that combines computational predictions with empirical data to facilitate the discovery and development of new antiviral agents. Our study is significant in its exploration of novel antiviral compounds and its approach to leveraging naturally occurring substances. HMOs, as components of human milk, present a unique opportunity to develop antiviral agents that are potentially safe and biocompatible. Furthermore, exploring the antiviral efficacy of HMOs contributes to a broader understanding of the therapeutic capabilities of human milk, extending beyond its known nutritional and immunological benefits. These findings could lead to further *in vitro* and *in vivo* studies validating the inhibitory effects of HMOs on viral proteins and aid in the development of novel broad-spectrum antiviral drugs or prophylaxes against respiratory viral infections.

## Results

### LNH and LNFP3 show higher binding affinities with viral proteins

Molecular docking analysis was conducted to determine the binding affinities of five representative HMOs, namely 2′-FL, LNnT, 3′-SL, LNH, and LNFP3, for four respiratory viral proteins, namely 1RUZ, 6VSB, 6BLH, and 5WB0. The results provide compelling insights into the potential role of these HMOs as viral protein inhibitors, possibly impeding viral attachment and the entry process (Table 1). A striking observation from the data is the high binding affinity of LNH for all four viral proteins studied, particularly for 1RUZ and 6VSB, for which the binding affinities were − 14 kcal/mol and − 15 kcal/mol, respectively. This strong interaction may be interpreted as effective viral inhibition. Similarly, LNFP3 demonstrated significant affinities, particularly for 1RUZ and 6VSB, further highlighting the potential of these HMOs as antiviral agents. In contrast, other HMOs, such as 2′-FL, LNnT, and 3′-SL, exhibited moderate affinities; their interactions were less pronounced than those of LNH and LNFP3, suggesting that these HMOs could be more effective at potentially blocking viral attachment to host cells. The molecular docking results for LNH and LNFP3 are intriguing when considering the complex nature of viral attachment mechanisms. The high binding affinity might indicate the ability of these HMOs to fit tightly into the binding pockets or interaction sites of the viral proteins, which could block the critical sites necessary for viral entry into host cells.

**Table 1:** Binding affinities (docking scores) of human milk oligosaccharides (HMOs) with different viral proteins.

HMOs	1RUZ (kcal/mol)	6VSB (kcal/mol)	6BLH (kcal/mol)	5WB0 (kcal/mol)
2′-FL	− 6.8	− 7.4	− 7.1	− 7
LNnT	− 8.1	− 7.8	− 6.7	− 6.6
3′-SL	− 7.2	− 7.4	− 6.2	− 6.8
LNH	− 14	− 15	− 12.5	− 11.4
LNFP3	− 12.8	− 11.4	− 10.1	− 10.6

**Figure 2:**
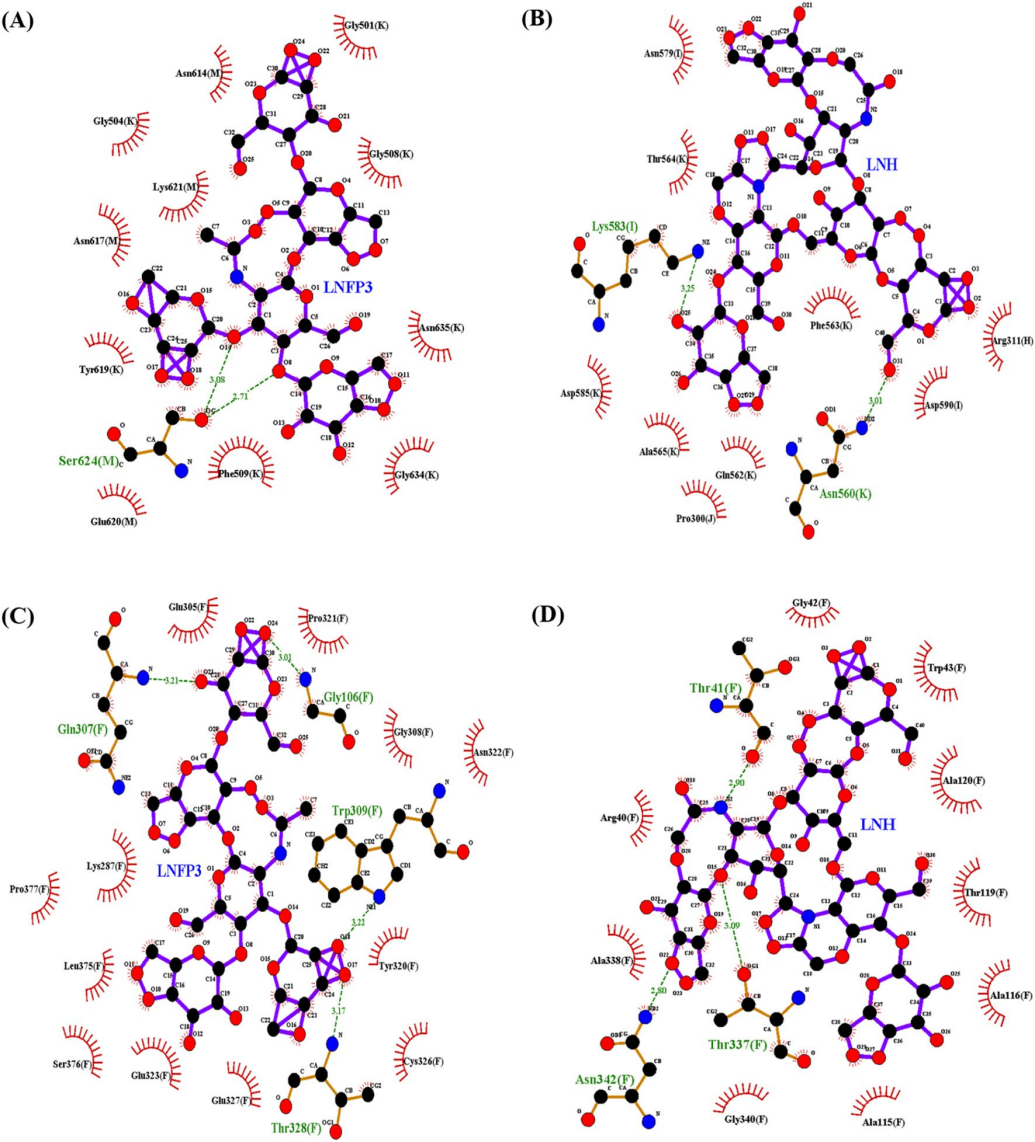

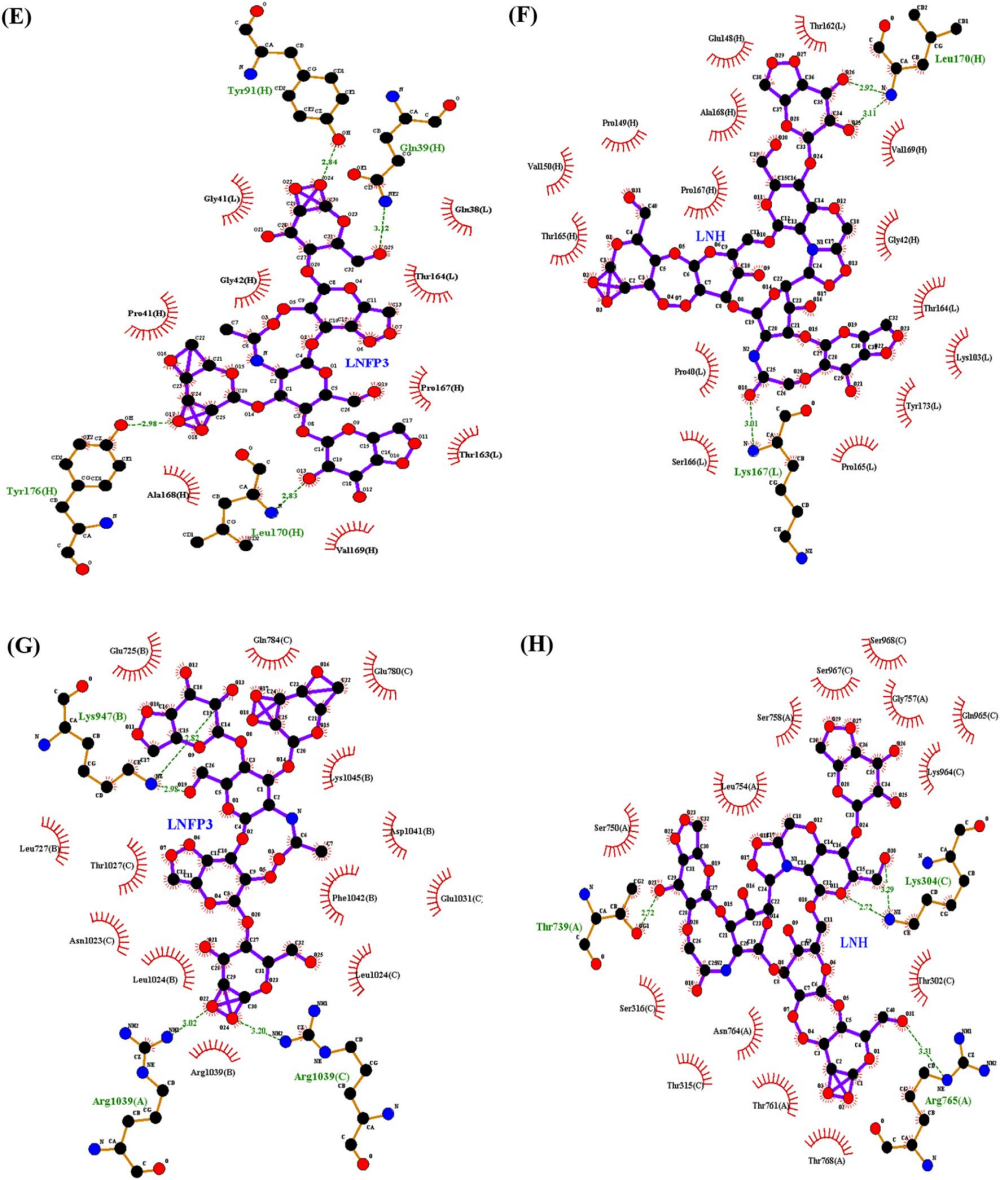
Ligand interaction maps for LNFP3 and LNH with different viral proteins. (**A**) 1RUZ-LNFP3, (**B**) 1RUZ-LNH, (**C**) 5WB0-LNFP3, (**D**) 5WB0-LNH, (**E**) 6BLH-LNFP3, (**F**) 6BLH-LNH, and (**G**) 6VSB-LNFP3, H. 6VSB-LNH. Each panel corresponds to a different protein–ligand complex detailing specific amino acid interactions such as H-bonds (dashed lines), hydrophobic interactions (arcs with spokes), and ionic interactions (arrows).

The molecular details of the interactions of LNH and LNFP3 with viral proteins were further visualized by LigPlot (Fig. 2), which revealed significant hydrogen bonding interactions indicative of potential inhibitory effects on these proteins. For the 1RUZ protein, the LNFP3-1RUZ complex exhibited two hydrogen bonds with Ser624, with bond lengths of 3.08 Å and 2.71 Å. In contrast, the LNH-1RUZ complex formed hydrogen bonds with Asn560 and Lys583, with bond lengths of 3.01 Å and 3.25 Å, respectively, suggesting that each HMO has a distinct binding modality. The LNFP3-5WB0 complex established four hydrogen bonds at Gly106, Gln307, Trp309, and Thr328, with bond lengths ranging from 3.03 Å to 3.22 Å. The LNH-5WB0 complex showed three hydrogen bonds at Thr41, Thr337, and Asn342, with bond lengths of 2.90 Å, 3.09 Å, and 2.80 Å, respectively, again highlighting that different HMOs maintain specific interaction patterns. The LNFP3-6BLH complex displayed four hydrogen bonds involving Gln39, Tyr91, Leu170, and Tyr176, with bond lengths between 2.83 Å and 3.12 Å, whereas the LNH-6BLH interaction was characterized by three hydrogen bonds at Lys167 and Leu170, with bond lengths of 3.01 Å, 2.92 Å, and 3.11 Å, respectively, indicating a unique binding orientation. Finally, the interaction between LNFP3 and the 6VSB protein resulted in four hydrogen bonds with two bonds at Lys947 (chain B) and one at Arg1039 (chain A) and Arg1039 (chain C), with bond lengths ranging from 2.82 Å to 3.20 Å. The LNH-6VSB complex made four hydrogen bonds with Lys304, Thr739, and Arg765, with bond lengths ranging from 2.72 Å to 3.31 Å, signifying a potentially robust interaction. These molecular docking results emphasize the ability of the HMOs, LNFP3 and LNH, to form specific and potentially inhibitory interactions with key respiratory viral proteins, thereby suggesting their promising role as antiviral agents.

### LNH and LNFP3 form more stable complexes with viral proteins

**Figure 3:**
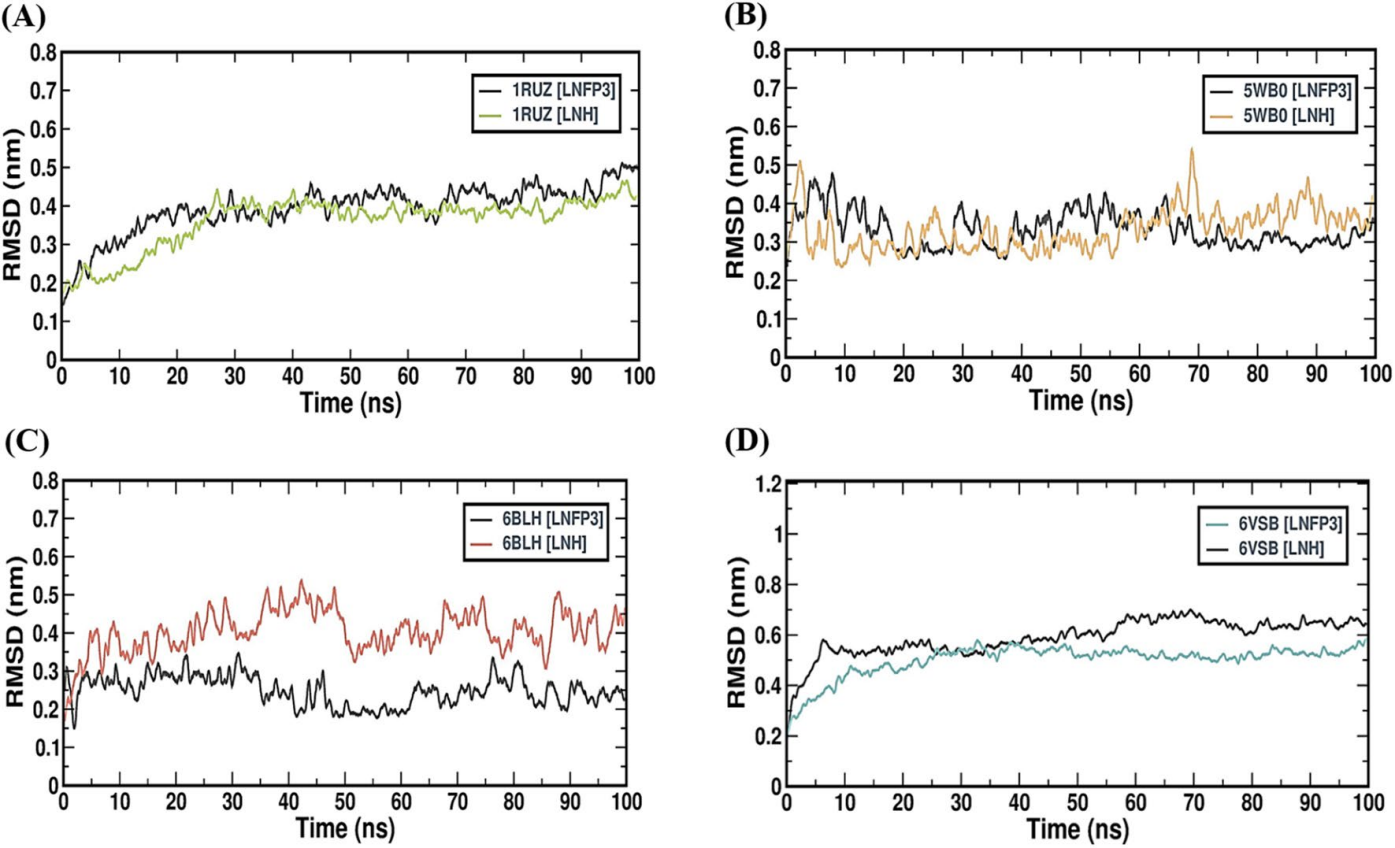
Comparative RMSD profiles of LNFP3 and LNH with different viral proteins. (**A**) 1RUZ-LNFP3 versus 1RUZ-LNH, (**B**) 5WB0-LNFP3 versus 5WB0-LNH, (**C**) 6BLH-LNFP3 versus 6BLH-LNH, and (**D**) 6VSB-LNFP3 versus 6VSB-LNH. RMSD values indicate differences in the conformational stability between the ligand-bound and unbound states.

Based on the results of molecular docking, LNH and LNFP3 were subjected to further MD simulations with all the chosen viral proteins using GROMACS, which supported the hypothesis that HMOs form stable complexes with key respiratory viral proteins. Root mean square deviation (RMSD) is a critical measure of the stability of protein-ligand complexes during MD simulations. The RMSD values of 1RUZ in complex with LNH and LNFP3 both plateaued off around 0.4 nm, suggesting that the interactions were stable throughout the 100 ns simulation period. The minor deviations observed for the 1RUZ-LNH complex in the initial 20 ns can be attributed to the system reaching equilibrium, after which the complex demonstrated a more stable RMSD profile (Fig. 3A). Both the 5WB0-LNH and 5WB0-LNFP3 complexes showed increased stability after an initial period of equilibration. Contrary to our initial analysis, the 5WB0-LNH complex demonstrated a marginally higher RMSD value than the 5WB0-LNFP3 complex after approximately 30 ns, suggesting a less stable interaction for the LNH complex (Fig. 3B). The 1RUZ and 5WB0 protein complexes with LNH and LNFP3 maintained relatively low and stable RMSD values indicating that the binding of these HMOs does not significantly disrupt the protein structure. These complexes are particularly promising for therapeutic purposes because they exhibit the stability needed to inhibit viral entry effectively. For the 6BLH-LNH and 6BLH-LNFP3 complexes, the RMSD values were around 0.3 nm, with the LNFP3 complex showing slightly greater stability. This could suggest tighter binding or less conformational change upon binding with LNFP3 (Fig. 3C). In general, complexes with the 6VSB protein showed higher RMSD values than those with other proteins, especially the 6VSB-LNH complex. This is indicative of a dynamic interaction, which could suggest a more flexible binding site or a larger conformational change upon ligand binding (Fig. 3D). The 6VSB complexes displayed higher RMSD values without a trend indicating loss of structural integrity. The elevated RMSD values could reflect the inherent flexibility of the spike protein’s receptor-binding domain, which is known to undergo conformational shifts during the viral infection cycle.

For all the complexes, the RMSD values reached a plateau early in the simulation period, which indicates stabilization of the protein-ligand complexes. Notably, none of the complexes exhibited a steadily increasing RMSD, which would have suggested a drift from the initial binding conformation and potential instability of the complexes. This analysis demonstrates that HMOs, particularly LNHs, can form stable complexes with various respiratory viral proteins, which could indicate their potential to disrupt viral entry mechanisms without compromising the integrity of protein structures. The data from the 6VSB complexes, despite showing higher RMSD values, do not necessarily negate the potential of HMOs. Instead, these findings may reflect the dynamic nature of the spike protein and warrant further investigation to determine the implications of these dynamics for HMO binding efficacy and potential antiviral properties.

### LNH and LNFP3 protein complexes share comparable rigidity

**Figure 4:**
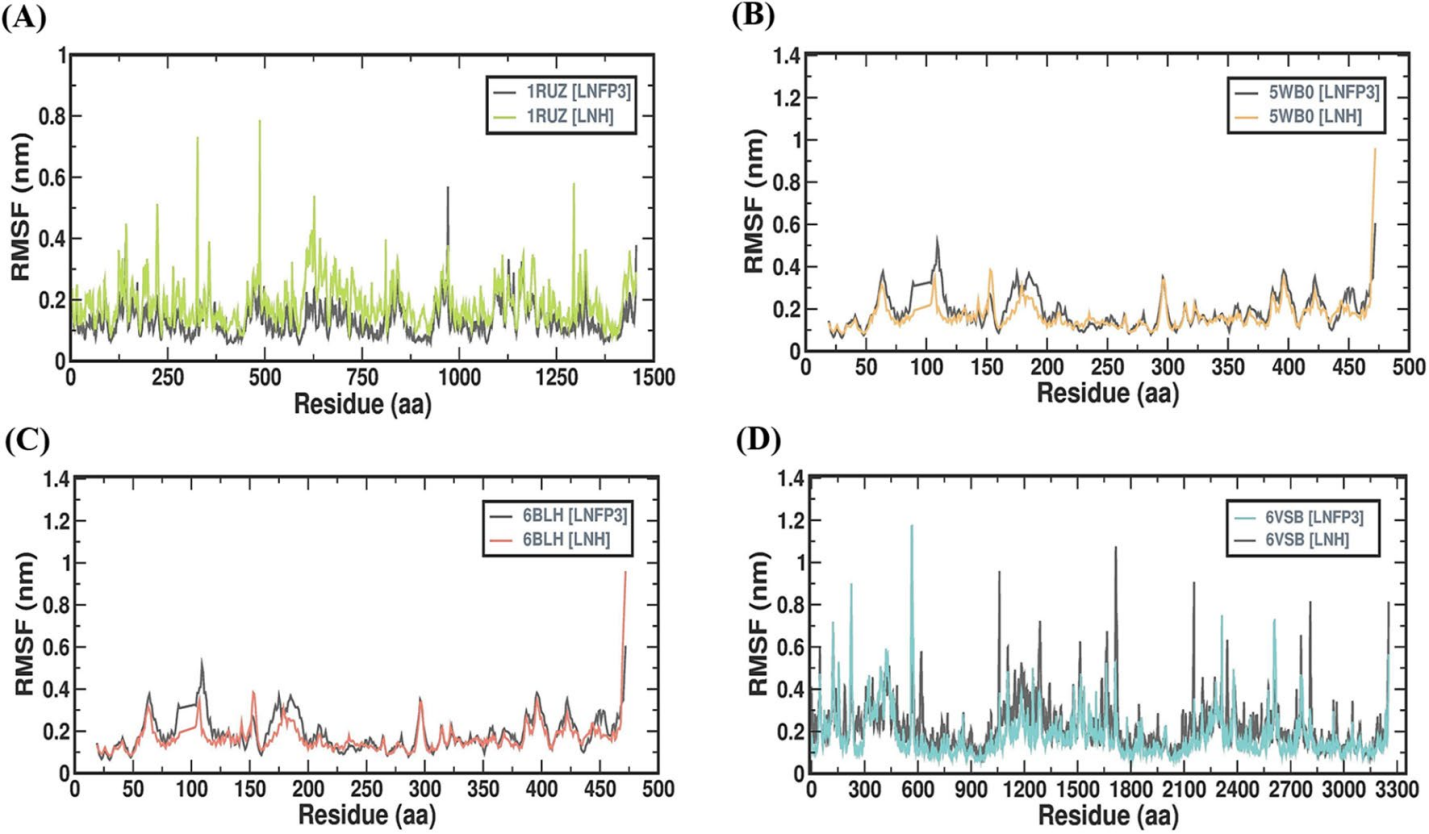
Comparative RMSF profiles of LNFP3 and LNH with different viral proteins. (**A**) 1RUZ-LNFP3 versus 1RUZ-LNH, (**B**) 5WB0-LNFP3 versus 5WB0-LNH, (**C**) 6BLH-LNFP3 versus 6BLH-LNH, and (**D**) 6VSB-LNFP3 versus 6VSB-LNH. Peaks indicate regions of higher flexibility or conformational changes upon ligand interaction.

Root mean square fluctuation (RMSF) provides a residue-by-residue analysis of protein flexibility, which is crucial for understanding the dynamic behavior of protein-ligand interactions. The RMSF profiles of complexes formed by the 1RUZ, 5WB0, 6BLH, and 6VSB proteins with LNH and LNFP3 HMOs were studied over a 100 ns simulation period (Fig. 4). The 1RUZ protein complexes revealed lower fluctuations across most residues when bound to both LNH and LNFP3, with certain regions exhibiting more flexibility. These RMSF peaks could correspond to loop regions or domains with inherently greater mobility and are not necessarily indicative of instability introduced by ligand binding. The 5WB0 complexes showed a similar trend, with overall low RMSF values, suggesting that the HMOs were stably bound. Notably, the 5WB0-LNH complex displayed slightly lower RMSF values across residues, suggesting that it is more rigid and potentially stable when compared to the 5WB0-LNFP3 and other complexes. The 1RUZ and 5WB0 complexes data demonstrated that the binding of HMOs did not induce significant fluctuations across amino acid residues, suggesting that the protein-ligand interactions are stable and do not disrupt the protein’s functional conformation. The fluctuations within the 6BLH complex, while slightly greater than those of the other complexes, remained within a stable range, which is encouraging for a potential therapeutic candidate. The RMSF values for the 6VSB protein displayed a distinct pattern with several pronounced peaks reflecting the protein’s larger size and the complex conformational dynamics of the spike protein, including its receptor-binding domain. Despite greater fluctuations, the presence of HMOs does not appear to introduce additional instability, suggesting that their binding does not adversely affect the conformational behavior of the protein. The RMSF analysis across the four viral protein complexes with LNH and LNFP3 suggested that the HMOs generally maintain stable interactions with the viral proteins, as evidenced by the lack of significant fluctuations upon ligand binding. The observed flexibility in certain protein complexes is consistent with the known dynamic regions of these proteins, which are crucial for their biological functions. These data indicate that the HMOs, particularly LNHs, may be effective in forming stable interactions with respiratory viral proteins, supporting their potential role as inhibitors. The specific regions of increased flexibility identified in the RMSF profiles may also provide targets for further investigation and potential drug development.

### LNH and LNFP3 protein complexes maintain structural compactness

**Figure 5:**
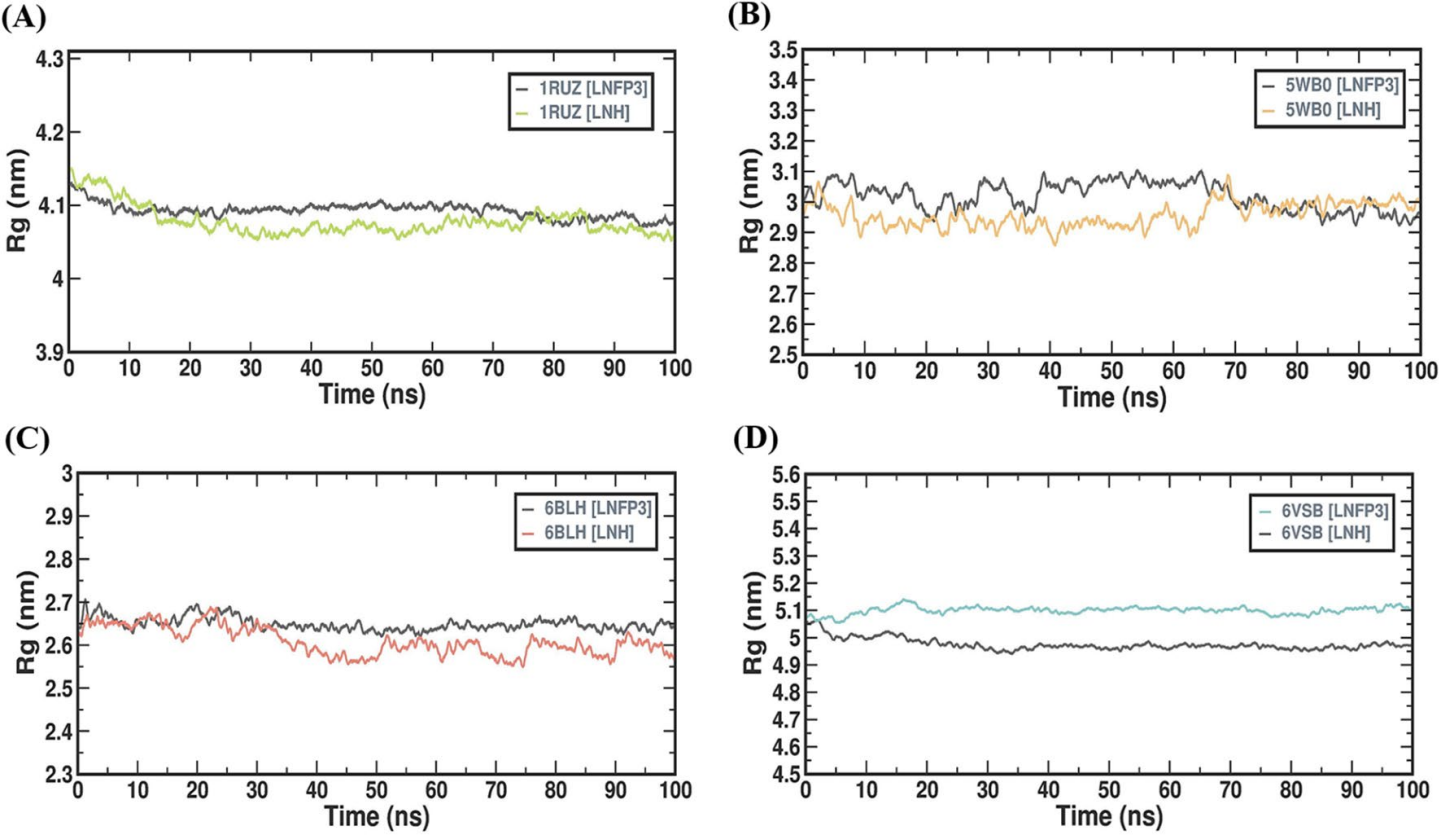
Radius of gyration (Rg) analysis for protein–ligand complexes over time. (**A**) 1RUZ-LNFP3 versus 1RUZ-LNH, (**B**) 5WB0-LNFP3 versus 5WB0-LNH, (**C**) 6BLH-LNFP3 versus 6BLH-LNH, and (**D**) 6VSB-LNFP3 versus 6VSB-LNH. The compactness of the protein structures observed during the MD simulations are shown.

The radius of gyration (Rg) gives an understanding of the compactness and folding behavior of a protein over time. The Rg was analyzed for protein-ligand complexes involving all four viral proteins and the HMOs, LNH and LNFP3 throughout a 100 ns MD simulation period. The Rg profiles of all the complexes studied demonstrated that the HMOs maintained consistent protein compactness and tertiary structures across the entire duration of the simulation (Fig. 5). The 1RUZ complexes with both LNH and LNFP3 showed consistently low variations in Rg values, suggesting that a tightly packed and stable structure was maintained throughout the simulation. The Rg values plateaued off around 4.1 nm, indicative of a stable tertiary structure, which is crucial for the potential inhibitory effect of the ligand. For the 5WB0 complexes, the Rg values exhibited minimal fluctuations, hovering at approximately 3.0 nm, which reflects a stable and compact structure upon HMO binding. The LNH and LNFP3 ligands appear to maintain the protein’s structural integrity, which is a usually desirable characteristic for potential inhibitors. The 6BLH complexes displayed an Rg profile with slight fluctuations, albeit within a narrow range, suggesting a stable ligand-induced conformation. The Rg values for complexes involving both the HMOs remained close to 2.6 nm, indicating a more consistent and stable protein structure, which is essential for the proper functioning of the viral attachment protein. The Rg profile of the 6VSB protein complexes reflected a slightly greater Rg value averaging about 5.0 nm, which suggests a less compact structure, which could be attributed to the inherent flexibility and larger size of the spike protein. Despite this, the Rg profiles of the 6VSB complexes with LNH and LNFP3 were stable, suggesting that the ligands maintained an interaction that did not compromise the overall structural stability even for dynamic proteins such as the spike glycoprotein. Rg analysis across the different protein-HMO complexes suggested that HMOs can effectively bind and still maintain the structural compactness necessary for the stability of protein complexes. A detailed analysis of the Rg profiles complements the RMSD and RMSF analyses by adding an additional layer of understanding regarding the structural stability of protein-ligand complexes. This reinforces the potential role of HMOs as stable inhibitors of viral proteins and supports their further investigation as antiviral agents.

### LNH and LNFP3 interact stably with large surfaces of 1RUZ and 6VSB

**Figure 6:**
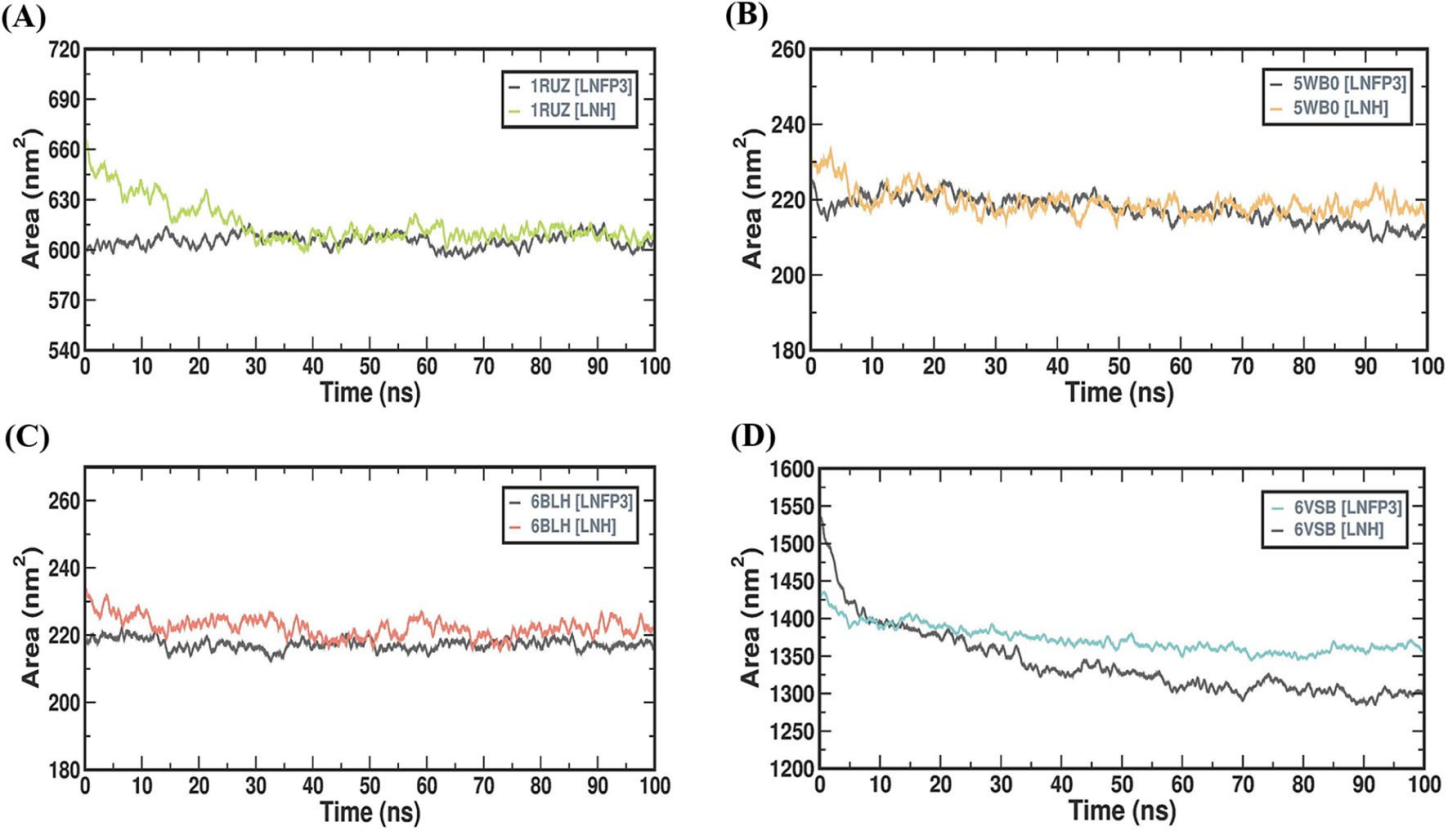
SASA trends for proteins in complex with LNFP3 and LNH ligands. (**A**) 1RUZ-LNFP3 versus 1RUZ-LNH, (**B**) 5WB0-LNFP3 versus 5WB0-LNH, (**C**) 6BLH-LNFP3 versus 6BLH-LNH, and (**D**) 6VSB-LNFP3 versus 6VSB-LNH. SASA values indicate the dynamic exposure of protein surfaces to the solvent when associated with different ligands.

The solvent accessible surface area (SASA) is a measure of the surface area of a protein that is accessible to a solvent, which reflects the exposure of hydrophobic and hydrophilic patches and the packing of the protein’s hydrophobic core. The SASA values provide vital information on how the binding of HMOs to viral proteins affects the proteins’ interactions with their environment (Fig. 6). The SASA profiles of the 1RUZ complexes revealed that the area remains relatively consistent throughout the 100 ns simulation period. The 1RUZ-LNH complex showed a slightly greater SASA, averaging about 615 nm^2^, while the 1RUZ-LNFP3 complex maintained an SASA close to 605 nm^2^. The consistency of SASA values suggests that ligand binding does not lead to significant exposure or burial of the protein’s hydrophobic core. For the 5WB0 complexes, the SASA profiles displayed a slight increase in the initial 10 ns, followed by stabilization. Both HMOs demonstrated similar SASA values, with the complexes averaging about 218 nm^2^, which indicates a stable surface area exposed to the solvent, suggesting stable protein-ligand interactions without significant unfolding or compaction. The 6BLH complexes exhibited low variability in SASA, indicating stable interactions over the entire simulation period. The SASA values for the 6BLH-LNH and 6BLH-LNFP3 complexes hovered around 220 nm^2^, further supporting the view of stable hydrophobic core packing upon ligand binding. The 6VSB complexes had higher SASA values, reflecting the larger surface area of the spike protein. Throughout the period of simulation, the SASA values fluctuated within a narrow range of 1340–1372 nm^2^ for the 6VSB-LNH and 6VSB-LNFP3 complexes, indicating that the protein consistently maintained its exposed surface area in the presence of both the HMOs. The SASA analysis across the protein-HMO complexes demonstrated that the HMOs maintained stable interactions with the viral proteins, as evidenced by the consistent SASA values throughout the simulation. This suggests that HMOs do not disrupt protein surface interactions with the solvent or destabilize the hydrophobic core. The SASA analyses corroborate other parameters by providing insights into protein surface characteristics in the presence of HMOs.

### LNH protein complexes accommodate robust hydrogen bonds

**Figure 7:**
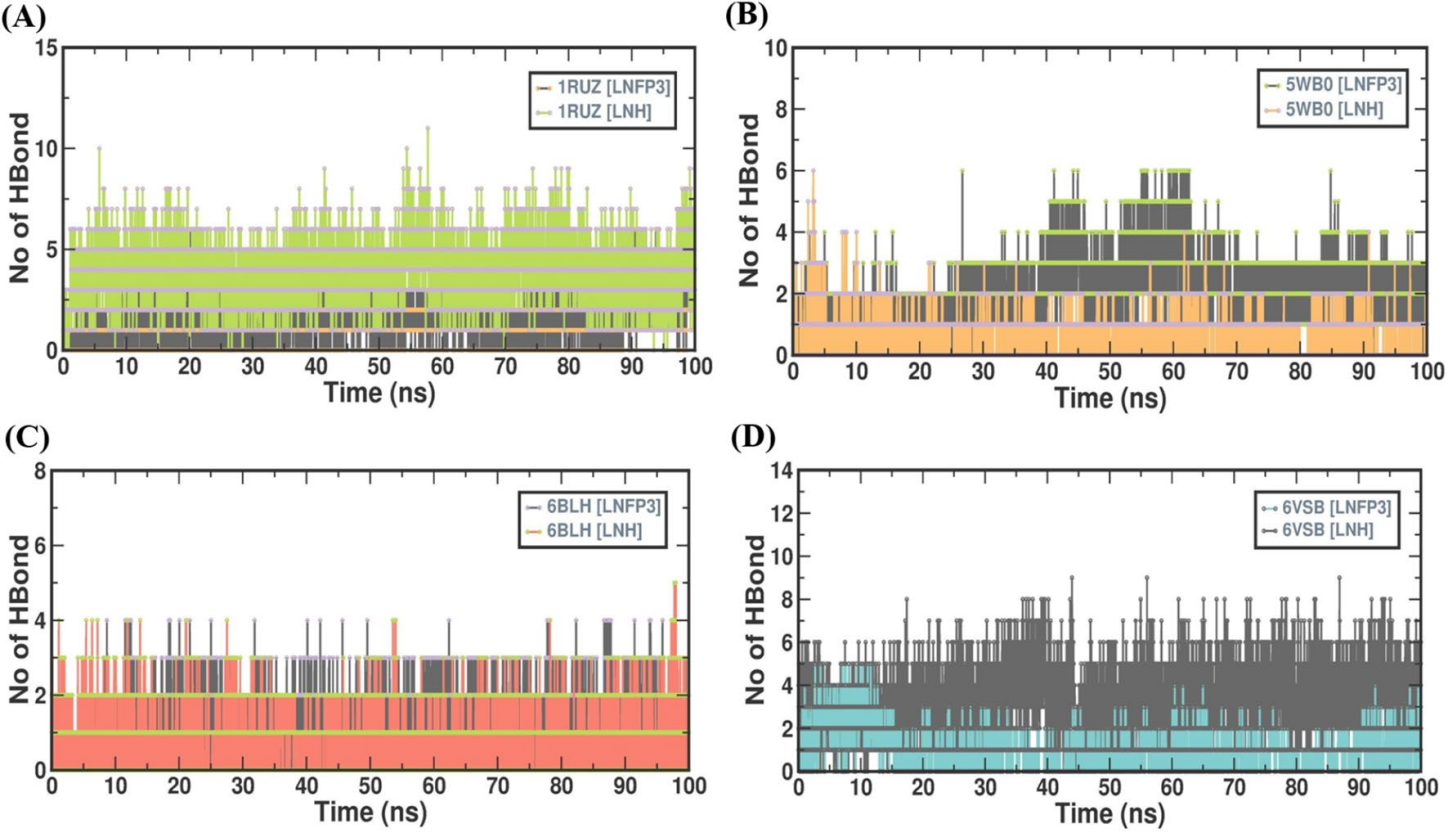
H-bond dynamics in protein–ligand interactions over time. (**A**) 1RUZ-LNFP3 versus 1RUZ-LNH, (**B**) 5WB0-LNFP3 versus 5WB0-LNH, (**C**) 6BLH-LNFP3 versus 6BLH-LNH, and (**D**) 6VSB-LNFP3 versus 6VSB-LNH. The graphs indicate the stability and variability of H-bond interactions within the protein–ligand complexes over time.

Hydrogen bonds are key determinants of the stability and specificity of protein-ligand interactions. This study analyzed the number of intramolecular hydrogen bonds between the selected viral proteins and the designated HMOs, LNH and LNFP3 through a 100 ns MD simulation period, which offered insights into their stability and potential efficacy (Fig. 7). The 1RUZ protein complex with LNH formed a consistently higher number of hydrogen bonds than with LNFP3, suggesting increased stability and binding affinity. Interestingly, in the case of the 5WB0 complex, LNFP3 showed a tendency to form a greater number of hydrogen bonds than LNH, indicating that different protein environments may influence the hydrogen bonding capacity and stability of the interactions with HMOs. This is consistent with the notion that a higher number of hydrogen bonds can contribute to more stable protein-ligand complexes. The 6BLH complexes maintained a stable number of hydrogen bonds, especially with LNH, further supporting the potential of HMOs as inhibitors that can stably bind to proteins over time. Despite the inherent flexibility observed in the 6VSB spike protein, the number of hydrogen bonds formed with the HMOs did not decrease, indicating that ligand binding does not destabilize the existing hydrogen bond network within the protein. The hydrogen bonding analyses across the protein–HMO complexes suggest that LNH may form more stable and potentially robust interactions with the viral proteins than LNFP3. The consistent number of hydrogen bonds observed for the LNH complexes indicates that it maintains stable interactions with the viral proteins, reinforcing its potential as a candidate for further investigation in therapeutic applications aimed at disrupting viral attachment and entry.

### LNH protein complexes reveal diverse conformational landscapes

**Figure 8:**
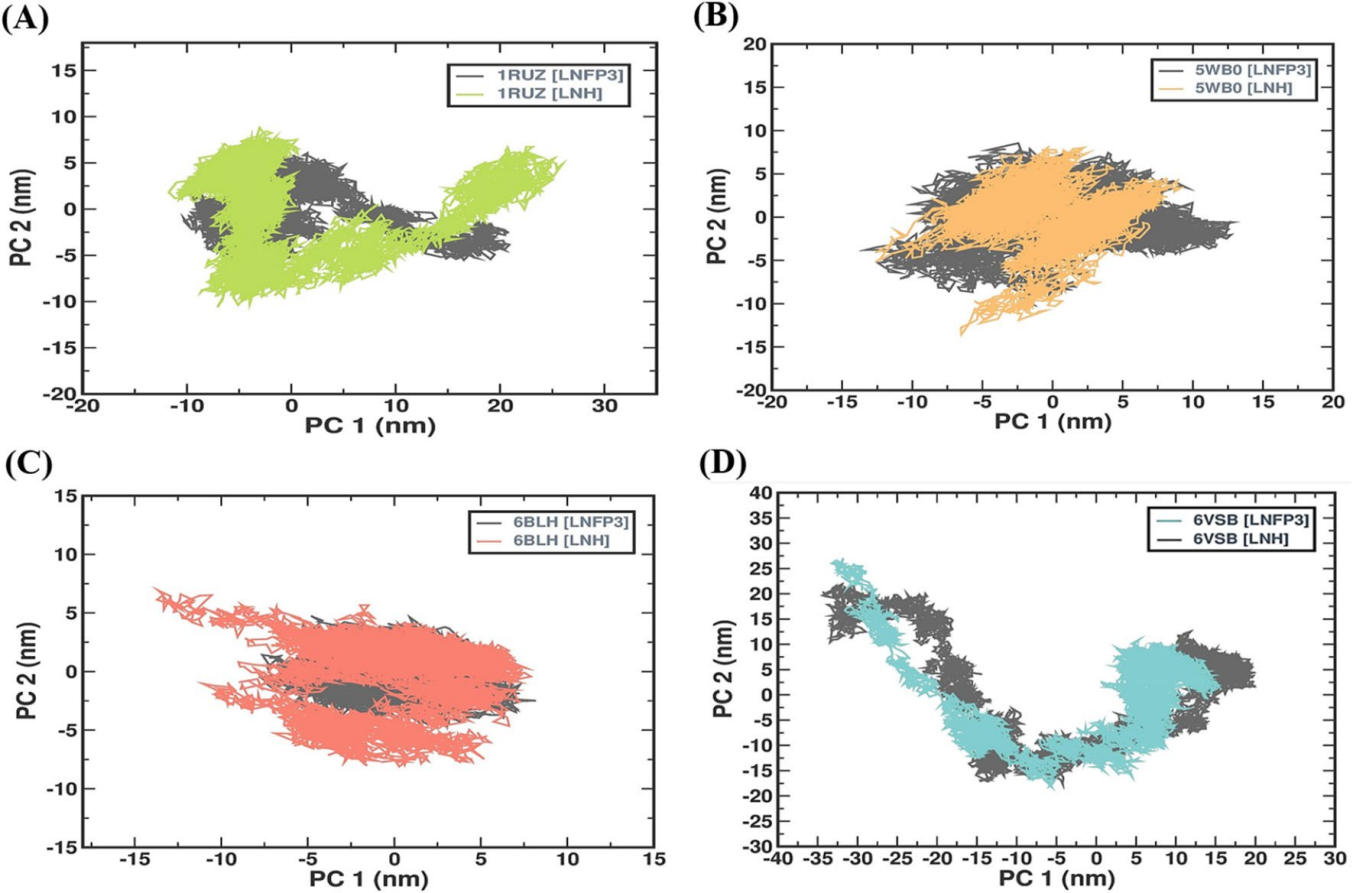
PCA of protein dynamics with LNFP3 and LNH ligands. (**A**) 1RUZ-LNFP3 versus 1RUZ-LNH, (**B**) 5WB0-LNFP3 versus 5WB0-LNH, (**C**) 6BLH-LNFP3 versus 6BLH-LNH, and (**D**) 6VSB-LNFP3 versus 6VSB-LNH. The plots are based on the first two principal components (PC1 and PC2), which capture most of the variance in the protein motion data.

Principal component analysis (PCA) is a statistical tool that is used to identify patterns in data and express them in such a way as to highlight their similarities and differences. In MD simulations, PCA is used to analyze conformational changes in proteins or protein-ligand complexes by reducing the dimensionality of the large datasets generated. The PCA results of the protein-HMO complexes provided a visual representation of the diversity and range of conformations that the protein-ligand complexes took during the simulations (Fig. 8). The PCA plot for the 1RUZ complexes with LNH and LNFP3 revealed two distinct clusters suggesting that each ligand guides the protein towards exploring different conformational spaces. Of note, the 1RUZ-LNH complex displayed a more confined conformational space, indicating that it has a more specific binding mode or a more stable protein-ligand complex than the 1RUZ-LNFP3 complex. For the 5WB0 complexes, the PCA plot shows overlapping conformational spaces for both LNH and LNFP3, with the 5WB0-LNH complex covering a slightly broader range. This overlap may suggest that the binding of both HMOs stabilizes similar protein conformations, with LNH potentially inducing additional subtle conformational changes. The PCA for 6BLH indicated considerable overlap in the conformational space between the LNH and LNFP3 complexes. However, the 6BLH-LNFP3 complex demonstrated a tighter clustering in the PCA plot than the 6BLH-LNH complex, which may suggest a more restrained range of conformational states for LNFP3. This tighter clustering could indicate that LNFP3 has the potential to stabilize certain conformational states of the protein. The 6VSB complexes with LNH and LNFP3 present a broad and scattered PCA distribution, in line with the expected flexibility of the spike protein. Notably, the 6VSB-LNH complex appears to induce a more dispersed range of conformations, which could indicate that the protein is journeying between various states upon ligand binding. The PCA results highlight the diverse conformational landscapes the viral protein-HMO complexes can explore. The distinct patterns observed for different complexes suggest that HMOs, particularly LNH, may influence viral protein dynamics, uniquely affecting viral binding and function. The PCA adds depth to the MD results by revealing the extent of conformational diversity and the potential impact of HMO binding on the structural dynamics of proteins. This emphasizes the importance of considering the stability of protein-ligand interactions and their ability to affect proteins’ functional conformational states.

## Discussion

In this study, we investigated the antiviral potential of selected HMOs against specific respiratory viral proteins, building upon and extending previous research findings in this field. Our focus on the chosen HMOs, particularly 2′-FL, LNnT, 3′-SL, LNH, and LNFP3, and their interactions with key respiratory viral proteins, such as 1RUZ, 6VSB, 6BLH, and 5WB0, adds new insight into the antiviral capabilities of HMOs. Our molecular docking and MD simulation results confirmed the strong binding affinities of LNH and LNFP3, which is consistent with previous studies demonstrating the inhibitory effects of HMOs on various viral targets. Günther et al. synthesized dendrimers conjugated with sialyllactoses that inhibited human and avian influenza virus strains, suggesting that these conjugates can block viral attachment to host cells^17^. Pandey et al. demonstrated that sialylated HMOs, particularly 3′-SL, exhibited antiviral activity against avian influenza viruses, with in vivo studies in chickens showing effective viral neutralization^18^. Zevgiti et al. designed sialic acid and sialyllactose glycoconjugates and found that they mimic influenza hemagglutinin receptors and are recognized by the virus, potentially blocking viral infection^19^. Yu et al. employed a shotgun glycan microarray from isolated HMOs and found that they were specifically recognized by human influenza viruses, suggesting that a decoy receptor mechanism can inhibit viral entry^20^. These studies have collectively demonstrated that HMOs and their derivatives act as competitive inhibitors blocking influenza virus binding to host receptors, thereby providing a foundation for developing HMO-based antiviral therapeutics. This study revealed high binding affinity and potential inhibitory interactions between HMOs and the hemagglutinin protein, suggesting that HMOs can act as competitive inhibitors of viral attachment.

For coronaviruses, Li et al. identified the sialic acid-binding function of the Middle East respiratory syndrome coronavirus (MERS-CoV) spike glycoprotein^21^. In addition to the known receptor, namely dipeptidyl peptidase 4 (DPP4), MERS-CoV also binds to sialic acid predominantly via α2,3-linkages. This binding is localized to the S1A subdomain of the spike protein, suggesting a role in the initial attachment phase, potentially influencing host range and tissue tropism. This discovery adds to our understanding of coronavirus entry mechanisms, particularly highlighting the significance of glycan interactions in viral attachment and infection processes. Recently, Yu et al. evaluated the antiviral activity of four HMOs, such as 3′-SL. 6′-SL, 2′-FL, and 3′-FL against the SARS-CoV-2 spike receptor-binding domain (RBD) in vitro^22^. Their findings indicate that 2′-FL and 3′-FL exhibit significant anti-binding activity against the RBD, with 2′-FL showing a more potent effect. This inhibition presumably occurs through a competitive binding mechanism, suggesting that HMOs could be explored as potential agents for preventing SARS-CoV-2 infections. Our study further extends these findings by showing that LNH and LNFP3 also exhibit high binding affinities for the spike protein of SARS-CoV-2 (6VSB), suggesting that they may function much like receptor mimics and prevent viral entry. While our study presents a comprehensive analysis of the binding affinity of HMOs to the spike protein of SARS-CoV-2, we recognize the dynamic nature of viral evolution, especially the rapid mutations observed in the RBD of the spike protein. The RBD is a critical determinant of host receptor recognition and thus a prime target for HMO binding as potential inhibitors. Given the structural diversity of HMOs, it is likely that certain HMOs may retain binding capacity across different strains of SARS-CoV-2 by mimicking the structural features of the host cell receptors that are conserved across strains. This mimicry may enable HMOs to bind to conserved regions of the spike protein’s RBD, which remain stable despite mutations in other regions. Our findings suggest that HMOs like LNH and LNFP3 exhibit strong binding affinities to the spike protein, which might translate to a broad-spectrum antiviral activity, potentially covering various strains. Nevertheless, the ability of HMOs to bind to new variants of the spike protein necessitates ongoing evaluation as new structural data becomes available. The broad-spectrum potential of HMOs, emphasized by their structural diversity and the conservation of certain host receptor features by the virus, provides a promising avenue for developing HMO-based interventions that could be effective against multiple strains of SARS-CoV-2.

Similarly, the study by Duska-McEwn et al. provides important insights into the role of 2′-FL and 3′-FL in reducing the RSV viral load in the airway epithelial cells through binding to glycoproteins^23^. HMOs are also known to inhibit many enteric viruses, such as noroviruses and rotaviruses, where they have been shown to mimic blood group antigens and secretory H type-1 antigens, respectively, interfering with viral capsid proteins and reducing infectivity^24–26^.

Furthermore, previous research on HIV has shown that specific glycans like LewisX can block the interaction between the HIV gp120 envelope protein and the dendritic cell-specific intercellular adhesion molecule-3 grabbing nonintegrin (DC-SIGN), highlighting a mechanism of action that could inhibit the transfer of HIV-1 on to CD4^+^ T lymphocytes^27^. These studies have laid the groundwork for understanding the mechanisms by which HMOs exert their antiviral effects, primarily through receptor decoy mechanisms. Our study not only confirms the potential of HMOs as broad-spectrum antiviral agents but also provides a detailed molecular basis for their interaction with respiratory viral proteins. These results align with and extend the current understanding of the biological functions of HMOs, particularly their role in antiviral defense. The significant binding affinities observed suggest that these HMOs can effectively bind to and potentially block key interaction sites on the viral proteins necessary for their cellular entry. The molecular docking results are further supported by MD simulations, which provide deeper insights into the stability and interaction dynamics of the HMO-protein complexes. Consistent RMSD values across all the complexes suggest that the binding of HMOs does not induce significant structural perturbations in the viral proteins, a desirable trait for potential inhibitors. This stability, as evidenced by RMSF and SASA values, indicates that HMOs maintain the structural integrity of the viral proteins upon binding, highlighting their potential as viable antiviral agents.

In conclusion, hydrogen bonding analysis revealed that LNHs made more stable and potentially stronger interactions with viral proteins than LNFP3s. These findings suggest a specific and consistent mode of interaction, which is critical for understanding how these natural compounds might impede viral attachment and entry. The PCA added a unique dimension to our findings, revealing the diverse conformational landscapes explored by viral protein-HMO complexes, which could influence the binding and function of these proteins. While molecular docking and MD simulations are sophisticated tools that can predict how HMOs might structurally attach to viral receptors, they should be viewed as part of a predictive framework that requires empirical validation. These methods provide valuable hypotheses on the potential binding modes and dynamics of HMO-receptor interactions, which must be corroborated by experimental data to confirm the actual binding configurations and affinities. Our study presents a computational exploration of the potential interactions between HMOs and viral receptors, laying the groundwork for future experimental investigations to validate and refine these predictions. Although LNH and LNFP3 demonstrated higher binding affinities to the viral proteins studied, suggesting their potential as efficient viral entry inhibitors, it is recognized that binding affinity is one of several factors that contribute to the efficacy of inhibition. The true measure of an HMO’s ability to prevent viral infection would be its performance in functional assays that mimic the viral entry process. It is also pertinent to consider the diversity of the HMO structures, which may confer different modes of antiviral action. For example, even if an HMO has a slightly lower binding affinity, it may still be a potent inhibitor if it can efficiently block a critical step in the viral entry process. Conversely, an HMO with a high binding affinity may not be as effective if it binds to non-critical sites that do not significantly impede the virus’s ability to infect the host cell. Even though, our in silico results are promising, we advocate for subsequent in vitro and in vivo assays to establish the functional inhibitory capabilities of LNH and LNFP3 in comparison to other structural HMOs such as 2′-FL, LNnT, and 3′-SL. These studies are crucial for determining the most effective HMOs among those tested for their potential applications in antiviral therapy.

The broader implications of this research are substantial in that identifying HMOs as potential inhibitors of key proteins of respiratory viruses opens up new avenues for developing natural, safe, and effective antiviral therapies. These initial findings pave the way for future in vitro and in vivo studies to corroborate the inhibitory effects of these HMOs on various viral proteins and support the development of novel broad-spectrum antiviral drugs or prophylaxis against respiratory viral infections. In the face of ongoing global health challenges posed by viral epidemics and pandemics, the potential role of HMOs in antiviral therapy represents a notable advancement in public health and infectious disease research.

## Methods

### Structure retrieval and preparation

The X-ray crystal structures of the selected respiratory viral protein targets, such as the 1918 influenza A virus H1 hemagglutinin (1RUZ), the human metapneumovirus fusion glycoprotein F0 (5WB0), the human respiratory syncytial virus central conserved region attachment glycoprotein (6BLH), and the 2019-nCoV (SARS-CoV-2) spike glycoprotein with a single receptor-binding domain (6VSB), were retrieved from the Protein Data Bank (PDB)^28^. The molecular structures of the five chosen HMOs, namely 2′-fucosyllactose (2′-FL) [170484], lacto-N-neotetraose (LNnT) [121853], 3′-sialyllactose (3′-SL) [124491031], lacto-N-hexaose (LNH) [124491500], and lacto-N-fucopentaose III (LNFP3) [124491136], were obtained from the PubChem database^29^. The protein and ligand structures were subsequently optimized and prepared for the docking studies by removing water molecules, heteroatoms, and other unnecessary residues using the PyMOL molecular visualization system^30^. The proteins were then subjected to energy minimization to remove steric clashes. Hydrogen atoms were added to the protein and ligand structures, and partial charges were assigned using the AMBER14 force field. The ligands (HMOs) were also optimized by adding hydrogen atoms and computing Gasteiger charges. Rotatable bonds were defined to allow flexibility during docking.

### Molecular docking

Molecular docking studies were performed using PyRx 0.8 (https://pyrx.sourceforge.io/; https://sourceforge.net/projects/pyrx/) and the AutoDock tool^31^. The Lamarckian Genetic Algorithm was employed. For each protein, a grid box was defined around the active site or the site of interest. The grid dimensions were set based on the proteins’ known or predicted interaction sites with cellular receptors. The HMOs were docked into the defined grid box of the selected proteins.

### MD simulations

MD simulations were performed using the GROMACS tool^32^ following docking studies of the protein-HMO complexes. The protein-ligand complexes were solvated in a cubic box with the TIP3P water model. Counterions were added to neutralize the system. The solvated systems were subjected to energy minimization using the steepest descent algorithm until the maximum force was less than 1,000 kJ/mol/nm. Two-phase equilibration was carried out with respect to NVT (constant number of particles, volume, and temperature) and NPT (constant number of particles, pressure, and temperature). NVT equilibration was performed at 300 K for 1 ns, followed by NPT equilibration at 1 atm for 1 ns. After equilibration, a 100-ns production MD run was executed for each system. The coordinates, velocities, and energy were saved every 10 ps for analysis. The MD trajectories were analyzed using the built-in GROMACS parameters, such as the root mean square deviation (RMSD), root mean square fluctuation (RMSF), radius of gyration (Rg), solvent accessible surface area (SASA), and hydrogen bond (H-bond), which were used to determine the stability and interactions in each simulated system.

## Data Availability

The datasets generated and/or analyzed during the study have been deposited in Zenodo which can be accessed via the link, 10.5281/zenodo.10440575.
